# Evaluation of Beef by Electronic Tongue System TS-5000Z: Flavor Assessment, Recognition and Chemical Compositions According to Its Correlation with Flavor

**DOI:** 10.1371/journal.pone.0137807

**Published:** 2015-09-14

**Authors:** Xinzhuang Zhang, Yawei Zhang, Qingxiang Meng, Ning Li, Liping Ren

**Affiliations:** 1 State Key Laboratory of Animal Nutrition, College of Animal Science and Technology, China Agricultural University, Beijing, 100193, China; 2 Ying Sheng Hengtai Technology co., LTD, Beijing, China; Wageningen UR Livestock Research, NETHERLANDS

## Abstract

The aim of this study was to assess the ability of electronic tongue system TS-5000Z to evaluate meat quality based on flavor assessment, recognition and correlation with the meat chemical composition. Meat was sampled from eighteen beef cattle including 6 Wagyu breed cattle, 6 Angus breed cattle and 6 Simmental breed cattle. Chemical composition including dry matter, crude protein, fat, ash, cholesterol and taurine and flavor of the meat were measured. The results showed that different breed cattle had different chemical compositions and flavor, which contains sourness, umami, saltiness, bitterness, astringency, aftertaste from astringency, aftertaste from bitterness and aftertaste from umami, respectively. A principal component analysis (PCA) showed an easily visible separation between different breeds of cattle and indicated that TS-5000Z made a rapid identification of different breeds of cattle. In addition, TS-5000Z seemed to be used to predict the chemical composition according to its correlation with the flavor. In conclusion, TS-5000Z would be used as a rapid analytical tool to evaluate the beef quality both qualitatively and quantitatively, based on flavor assessment, recognition and chemical composition according to its correlation with flavor.

## Introduction

Chemical compositions and physical indicators of meat are important criteria used to evaluate the meat quality in meat science. Modern consumers are increasingly concerned about production of safe meat with good palatability and high chemical composition quality [1]. The major parameters considered in the assessment of meat quality are appearance, juiciness, tenderness, flavor and chemical compositions. Flavor is a very complex attribute of meat palatability and the most important factor affecting consumers’ meat buying habits and preferences [2]. Chemical composition is an important factor directly influencing meat taste or flavor, such as intramuscular fat, free fatty acids, fatty acids or amino acid compositions. In addition, the chemical composition, most often the fat content or lean/fat ratio determines the meat price in the meat processing market [3]. However, many factors such as breed, gender, feeding system and slaughter procedure could influence the chemical composition of meat. Especially, many types of ground meat like hamburgers, patties and sausages with little visual difference has large different on chemical composition. Thus, there is a need to evaluate the chemical composition to ensure that the consumers get the right quality.

Human taste panels are preferred and are commonly used as a taste assessment method to evaluate appearance, juiciness, tenderness, flavor and acceptability of meat. However, high variability and subjectivity of the human sense of taste, as well as high cost and time consuming all limit the application of the human taste panels [4]. Another important shortcoming is human taste panels could not analyze the correlation between the flavor and chemical composition of meat. Particular interest for taste prediction by chemical compositions has therefore led to the development of alternative methods that address the difficulties associated with human panel test.

One tool to overcome these drawbacks of human taste panels is the so-called electronic tongue sensor systems. Electronic tongues are sensor array based robotic systems and the results are shown to have good reproducibility with low detection limits and high sensitivity [5]. The electronic tongue was applied as a rapid and low-cost method to quantitative and qualitative analysis of numerous foodstuffs during the last decade [6]. Applications of electronic tongues in food analysis have been done to compare the qualities of wine, tea and beer [7]. In recent years, the use of electronic tongues systems in the area of pharmaceutical development has also attracted more interests [8].

Electronic tongue sensor system TS-5000Z, which employs the same mechanism as that of the human tongue, converts the taste of various substances into numerical data. The electronic tongue sensor system can be applied to food flavor assessment because these results are correlated to the marks of taste panels. On the other hand the electronic tongue also can be used for multivariate analysis and the content of the most important food nutrients [9]. The aim of the present study is to assess the ability of electronic tongue sensor system TS-5000Z as a rapid analytical tool to evaluate the beef quality through flavor assessment, recognition and chemical compositions according to its correlation with flavor.

## Material and Methods

### Meat sampling procedure

This study was carried out in strict accordance with the recommendations in the Guide for the Care and Use of Laboratory Animals of the National Institutes of Health of China. The protocol was approved by the Animal Welfare Committee of China Agricultural University (Permit Number: DK1008). All surgeries were performed under sodium pentobarbital anesthesia, and all efforts were made to minimize suffering. Eighteen steers from three different cattle breeds: 6 Wagyu (carcass weight, 383±23kg; 28 months-old), 6 Angus(carcass weight, 350±28kg; 24 months-old), 6 Simmental(carcass weight, 293±17kg; 18 months-old) were used in this study. All animals were slaughtered in local commercial abattoir. After ageing at 1–4°C for 72 h, the longissimus lumborum (LL) was excised of each left half side and was cut between the 12th and 13th rib. The meat samples were minced twice through a 5 mm plate using a meat mincer (model JYS-A800, Joyoung, China) after removing the connective tissue and subcutaneous fat. Fifty grams of raw ground meat from each animal was weighed and prepared for measurement of the flavor by the electronic tongue sensor system TS-5000Z. The remainder of each meat sample was made into lyophilized powder for subsequent analysis of chemical compositions.

### Meat chemical composition analysis

Moisture, protein, fat, and ash were measured according to the AOAC (1999) [10]. Cholesterol concentration of LL was analyzed as described by Rule et al (2002) [11] using gas chromatography. Briefly, cholesterol was extracted with 3 ml of ethanol per 100 mg of dried tissue. The lipids were saponified by the addition of 1 ml of 33% (w/v) KOH and heating for 1hour in an 85–95°C water bath. Cholesterol was isolated on 6890N gas chromatograph equipped with an on-column capillary injector and a FID detector(Agilent Technologies, U.S.A.), which installed with a HP-5, 30 m × 0.32 mm inner diameter× 0.25 μm film column with injector temperature at 280°C and injector temperatures at 290°C. Samples (1.0μL) were injected into a split injection port. The initial oven temperature was 200°C and the temperature was then increased to 280°C at a ratio at 30°C/min, and remained isothermally at 280°C for 10 min afterward. The flow rate of gas and hydrogen flow rate is 350ml/min and 30ml/min respectively. Stigmasterol (NU-CHEK PREP, INC, U.S.A, purity>99%) was used as the internal standard to quantify the total cholesterol. The cholesterol content was calculated by peak area with reference to standard solution.

Taurine was determined by pre-column high performance liquid chromatography (HPLC) using 5—dimethyl amino naphthalene—1—sulfonyl chloride as derivatization agent according to Inoue et al (2003) [12] with minor modifications. Sample preparation: 1.00g meat sample (lyophilized powder) was weighed and mixed in 80ml distilled water at 60°C. After 10min, 1ml precipitator potassium ferrocyanide solution (15g/100ml) and 1ml zinc acetate solution (30g/100ml) was added. After being completely mixed, water was added to bring the final volume to 100ml. Finally, the mixture was centrifuged at 3035 × g (DL-6000B, Shanghai Anting, China) for 10min. The supernatant was stabilized for 24 h at 4°C in the dark condition and prepared to the derivatization. 1.0ml supernatant mixed thoroughly with 1.0ml Sodium carbonate buffer (80 mM, pH 9.5) and 1.0ml dansyl chloride solution (1.5 mg/mL acetonitrile). After 2h derivative reaction in darkness, 0.1ml Methylamine hydrochloride (20 mg/mL) was added to terminate the reaction. The supernatant was removed and clarified through a 0.45.m Whatman glass microfiber filter (Whatman, UK). An aliquot of the supernatant (20μL) was subjected to HPLC. Instrumental conditions: the derivation was detected by C_18_ reversed phase HPLC (Agilent 1100 series). A ZORBAX SB-C18 (4.6 x 150mm x 3.5mm) was used with an elution system with the ratio of 70 percent (A) sodium acetate buffer (10mM) and 30 percent (B) acetonitrile. The flow rate was 1ml/min. The fluorescence intensities were monitored at excitation and emission wavelengths of 330 and 530nm, respectively.

### Electronic tongue sensor system TS-5000Z

A taste sensor is required to exhibit global selectivity so that it responds consistently to the same taste similarly to the human tongue. After years of research with Prof. Toko's group at Kyushu University, Japan, they have successfully developed taste sensors based on an artificial lipid membrane that consistently responds to similar taste to the human tongue. All measurements were performed using the latest model electronic tongue sensor system TS-5000Z (Insent Inc., Japan). The response principle: The lipid in the taste sensor interacts with various taste materials via electrostatic and hydrophobic interactions, which causes a change in potential of lipid membrane. The change is detected by a computer to provide a sensor output. The underlying measurement principle is potentiometric and sensor responses are obtained as mV values consequently. According to the Nernst or Nikolsky-Eisenmann equation, the response of potentiometric chemical sensors depends logarithmically on the activity of the substance. The formula was described according to Guhmann et al (2012) [13].

#### Sensors

TS-5000Z was equipped with five lipid membrane sensors indicating different taste qualities and three corresponding reference electrodes. There are bitterness sensor (SB2C00), gustatory stimuli umami sensor (SB2AAE), saltiness sensor (SB2CT0), sourness sensor (SB2CA0), and astringency sensor (SB2AE1). 0.2 ml inner solution, which consists of 3.33 M potassium chloride in saturated silver chloride solution and is provided by Insent Inc (Atsugi-shi, Japan), was filled into each sensor prior to the beginning of experiments. The reference electrode was completely filled up with inner solution. All sensors were preconditioned in standard solution for one day before measurement.

#### Preparation of standard, washing and sample solutions

A standard solution serving as cleaning and reference solution was prepared by dissolving 30 mM potassium chloride and 0.3 mM tartaric acid in distilled water. Two washing solutions for negatively and positively charged sensors respectively were made by diluting absolute ethanol to ethanol 30% with distilled water and adding 100 mM hydrochloric acid for the negatively charged sensors or 100 mM potassium chloride and 10 mM potassium hydroxide for the positively charged sensors. All chemicals used were of analytical grade.

The water-soluble extraction (WSE) of meat was obtained according to the following procedures: 50.00g raw ground meat sample from each cattle was mixed with 200ml distilled water, and then homogenized for 1min using a refiner (FJ-200, Jintan, China). Next, the mixture was centrifuged at 1821 × g (DL-6000B, Shanghai Anting, China) for 10 min at 4°C. The supernatant was removed and filtered by a 0.22μm Whatman glass microfiber filter (Whatman, UK) to remove the excess solid for the analysis used taste sensing system TS-5000Z.

#### Electronic tongue system and measurement setup

A sensor check was conducted routinely before every measurement in order to assure that sensors were stable in the correct mV range. One measurement cycle consisted of measuring a reference solution (Vr), afterwards the sample solution (Vs), a short (2 × 3 s) cleaning procedure and measurement of the aftertaste (Vr^’^) followed by a cleaning procedure for 330s. The aftertaste was measured by determining the change of membrane potential caused by the substance adsorption to the lipid membrane after the short cleaning procedure. Both sensor output for taste (R), and sensor output for aftertaste, also called CPA value (change of membrane potential caused by adsorption), were calculated in relation to the preliminary determined sensor response to the reference solution (Vr). The formula was showed: R = Vs−Vr; CPA = Vr^’^−Vr.

The whole measurement procedure was performed for all samples and repeated afterwards up to four times. For further data treatment the first run was discarded as recommended by the supplier in order to enable conditioning of the sensors. The method was validated and described in previous paper Woertz et al (2011) [14]. Results were expressed as raw data in mV of the sample relative measurement to the reference. Data collected by electronic tongue were reviewed and the mean values of the last three cycles were used for statistical analysis.

## Statistical Analysis

The means of flavor values of the last three cycles were processed by Excel 2007 (Microsoft, USA). The meat chemical compositions and flavor assessment of different breeds of cattle were subjected to ANOVA of generalized linear model (GLM) procedures of SAS 9.0 (SAS Inst. Inc., Cary, NC). The chemical composition and flavor differences between breeds were analysed in terms of least square group means using Duncan’s multiple comparison tests. The means of flavor values of the last three cycles were used to identify different breeds of beef cattle by princomp model procedures of SAS 9.0 (SAS Inst. Inc., Cary, NC). Ignoring the effect of the breed, Pearson correlation coefficients and regression formula between the flavor and chemical composition were determined using CORR and REG model procedure of SAS 9.0 (SAS Inst. Inc., Cary, NC). Levels of significance were considered significant at *P* < 0.05, tendency at 0.05 < *P* < 0.1, and not significant at *P* > 0.1.

## Results and Discussions

### Chemical composition of different breed beef

Beef quality is influenced by many factors which can be divided for simplicity into two categories: factors directly linked with the animal (breed, age, sex, etc.) and factors external to the animal (diet, weather, slaughtering procedures, etc.) [15]. Table 1 showed that chemical composition was significantly influenced by cattle breed (*P*<0.05). The proportion of dry matter was significantly lower in Simmental than the other two cattle breeds (*P*<0.05), whereas there was no significant difference between Wagyu and Angus. The fat content of Wagyu and Angus was significantly higher than Simmental (*P*<0.05) in the present study which is in line with the notion that Wagyu and Angus cattle are found for their genetic predisposition to intense marbling [16]. On the contrary, the highest content of crude protein was found in Simmental, the lowest in Wagyu, Angus was intermediate, which was similar to the results reported by Xie et al (2012)[17]. The proportion of ash was significantly higher in Simmental than the other two cattle breeds (*P*<0.05), whereas no significant difference between the latter. The cholesterol content was also significantly influenced by cattle breed, with the highest level in Angus, intermediate in Wagyu, and lowest in Simmental. The taurine content in Angus was significantly higher than in Wagyu (*P*<0.05) and both Angus and Wagyu had no significant difference compared to Simmental (*P*>0.05).

**Table 1:** Chemical composition of different breeds of beef.

Item	Wagyu	Angus	Simmental	SEM	*P*
Moisture%	69.24^b^	67.50^b^	73.18^a^	0.7092	0.0001
Fat%(DM)	35.88^a^	25.69^b^	12.19 ^c^	1.6467	<0.001
Crude protein% (DM)	59.49^c^	69.58^b^	82.04^a^	1.6584	<0.001
Ash% (DM)	2.97^b^	3.26^b^	5.00 ^a^	0.4128	0.0008
Cholesterol (mg/100gDM)	101.45^b^	151.26^c^	61.29^d^	5.4087	<0.001
Taurine (mg/100g DM)	126.42^b^	179.67^a^	164.49 ^ab^	14.8120	<0.001

Note: Means in the same row with different superscripts are significantly different (*P*<0.05).

### Beef flavor assessment from different breeds of cattle

**Fig 1:**
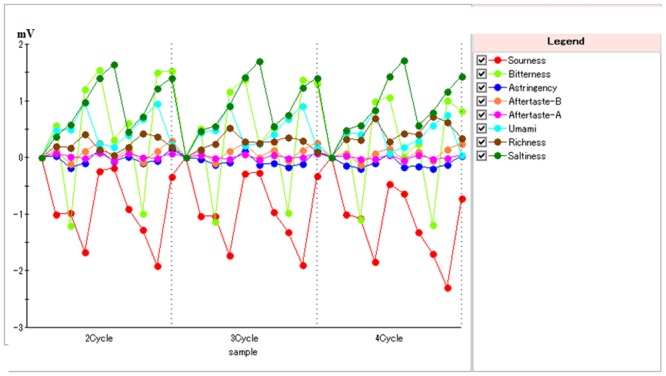
The stability of sensors response to various taste materials in the last three cycles of meat extract sample. Richness is Aftertaste from Umami.

Fig 1 showed the last three cycles of sensors response to various taste materials in meat extract sample. The first run was discarded as recommended by the supplier in order to enable conditioning of the sensors. As shown in Fig 1, all sensor responses were stability in cycles, thus the variation of sensor responses was minimal and that reproducible results could be trusted.

**Fig 2:**
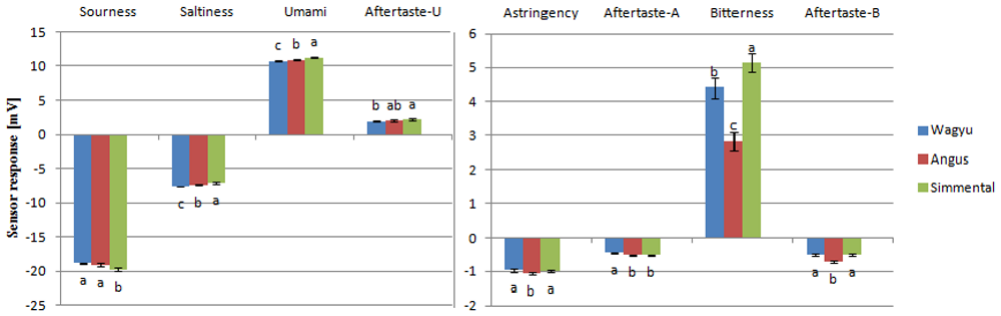
Flavor assessment of different breed beef.

Beef flavor is determined by its chemical compositions and influenced by the complex interactions between various chemical compounds of meat [2]. Taste is one component of flavor and an important determinant of the overall eating and drinking experience [18]. Therefore, different chemical composition resulted in differences flavor values, which are presented in Fig 2. Simmental cattle had the lowest sourness compared to other two breeds, whereas umami of Simmental cattle was higher than Angus, which was higher than Wangyu (*P*<0.05). Rank from high to low of saltiness was Simmental, Angus and Wagyu respectively (*P*<0.05). The most bitter breed was Simmental, followed by Wagyu, then Angus (*P*<0.05). The astringency values in Simmental and Wagyu were higher than Angus (*P*<0.05). Angus had lower aftertaste-B value than Wagyu and Simmental (*P*<0.05). The aftertaste-A of Wagyu was higher than Angus and Simmental (*P*<0.05). Aftertaste-U of Simmental was significantly higher Wagyu (*P*<0.05) and no significant difference between Angus and the other two breeds (*P*>0.05).

### Relationships between flavor and chemical compositions

The correlation coefficients between flavor and chemical composition was presented in Table 2. Fat content had the highest positive correlation with sourness(r = 0.8002, *P*<0.001) while was negatively correlated with umami (r = −0.9086, *P*<0.001) and saltiness (r = −0.8197, *P*<0.001). Sourness serves as a signal that warns animals against ingesting spoiled foods [19]. Fischer and Noble (1994) [20] reported that sourness increased with the decreasing pH, thus the highest positive correlation between fat and sourness could be interpreted by that the accumulation of organic acids produced by fat oxidation lead to the decreasing pH [21].

**Table 2:** The Pearson correlation coefficients between flavor and chemical composition.

Items	Sourness	Bitterness	Astringency	Aftertaste-B	Aftertaste-A	Umami	Aftertaste-U	Saltiness
Fat	0.8002^z^	-0.4390	-0.0866	-0.1868	0.5544^x^	-0.9086^z^	-0.3759	-0.8197^z^
Crude protein	-0.4157	-0.2485	-0.2736	-0.4908^x^	-0.7359^z^	0.7286^z^	0.4307	0.4029
Ash	-0.4417	0.2690	0.0856	0.1015	-0.4364	0.5916^y^	0.3579	0.7794^z^
Cholesterol	0.6473^y^	-0.9457^z^	-0.3539	-0.8615^z^	-0.0023	-0.5792^x^	-0.2642	-0.5736^x^
Taurine	-0.1017	-0.5066^x^	-0.2949	-0.4801^x^	-0.3946	0.0487	0.4454	0.0102

Levels of significance:

^x^ *P* < 0.05;

^y^ *P* < 0.01;

^z^ *P* < 0.001.

Umami is a Japanese concept meaning savory or delicious and is elicited by two types of chemical compounds: amino acids, such as monosodium glutamate (MSG) and aspartate, and purine nucleotides, such as inosine monophosphate (IMP) and guanosine monophosphate (GMP) [22]. For crude protein, the highest positive correlation was umami (r = 0.7286, *P*<0.001) and the aftertaste-A (r = −0.7359, *P*<0.001) and aftertaste-B (r = −0.4908, *P*<0.05) were negatively correlated. Therefore, the highest crude protein content of Simmental presented the highest umami value, while Wagyu with the lowest crude protein level had the lowest umami value.

Ash correlated positively with umami (r = 0.5916, *P*<0.01) and saltiness (r = 0.7794, *P*<0.001). Chabanet et al (2013) [23] suggested that a reduction in the salt content in foods may therefore have negative impact on their saltiness intensity. The breed with higher ash content presented higher saltiness intensity because ash contained many mineral salts.

Cholesterol correlated positively with sourness(r = 0.6473, *P*<0.01) and correlated inversely with bitterness (r = −0.9457, *P*<0.001), Aftertaste-B (r = −0.8615, *P*<0.001), umami (r = −0.5792, *P*<0.05) and saltiness (r = −0.5736, *P*<0.05). However, the correlation between cholesterol and taste sensing values only presented in numerical rather than causal relationship because cholesterol could not be soluble in water.

Taurine (2-aminoethane sulphonic acid) is a sulphur-containing amino acid in muscle as a free acid and has been linked with many biological actions include membrane stabilization, detoxification, antioxidation, osmoregulation, maintenance of calcium homeostasis, and stimulation of glycolysis and glycogenesis [24]. Bitterness (r = −0.5066, *P*<0.05) and Aftertaste-B (r = −0.4801, *P*<0.05) showed negative correlation with taurine. Zhang et al (2004) found that supplements of a daily dose of 6 grams (2 grams three times a day) taurine powder (Taisho, Tokyo, Japan) for 7 days may attenuate exercise-induced DNA damage and enhance the capacity of exercise due to its cellular protective properties [25]. Therefore, Angus had lower bitterness level and was possibly a good choice for human health as it has the highest levels of taurine content according to the present study.

Table 3 showed the regression formula between chemical composition and flavor. According to Table 3, chemical composition could be predicted by its high correlation flavor traits. Therefore, the results indicated that flavor had a good prediction to chemical composition.

**Table 3:** The regression formula between chemical composition and flavor.

y	X	R^2^	*P*
Fat	85.66–7.47 Umami	0.8256	<0.001
Protein	-2.09–25.21 Aftertaste-A	0.5415	<0.001
Ash	1.92+0.19 Saltiness	0.6075	0.0002
Cholesterol	43.81–6.71Bitterness	0.8944	<0.001
Taurine	-2.15–3.96 Bitterness+20.38 Aftertaste-U	0.5052	0.0051

Levels of significance: *P* < 0.05

### PCA score plot to discriminate different breeds of beef

**Fig 3:**
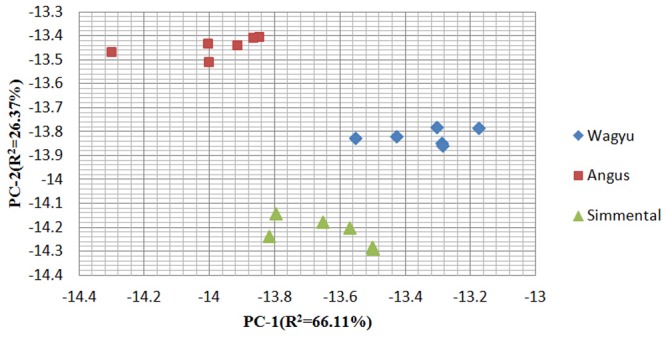
PCA score plot of different breed beef. The mean flavor values of the last three cycles were processed by PCA, which include sourness, umami, saltiness, bitterness, astringency, Aftertaste-B, Aftertaste-A and Aftertaste-U.

In order to better visualize the results, a principal component analysis (PCA) was carried out and the results are presented in Fig 3. The PCA map additionally exhibited the differences among different breeds according to the flavor of sourness, umami, saltiness, bitterness, astringency, Aftertaste-B, Aftertaste-A and Aftertaste-U. The main variance was explained by the first component, the x-axis (PC-1), and the smaller part of the variance covered by the second principal component was shown on the y-axis (PC-2). PC-1 represented about 66% of the total information, 26% of the information was displayed by PC-2. The eigenvalues of PC-1 and PC-2 were 5.29 and 2.11 respectively. Therefore, 92% differentiation between the samples was carried out with respect to PC-1, with additional information gained by PC-2. In addition, eigenvalues of the flavor traits within the principal components are listed in Table 4. Sourness, bitterness, astringency, Aftertaste-B, Aftertaste-A and saltiness would increase with increasing PC-1, whereas umami and Aftertaste-U would decrease. The highest correlation flavor trait to PC-1 was astringency. Similarity, PC-2 also had six positive flavor traits, including umami, saltiness, bitterness, astringency, Aftertaste-A and Aftertaste-U, and the rest of two negative flavor traits including sourness and Aftertaste-B. Bitterness had a highest positive correlation with PC-2.

**Table 4:** Eigenvalues of the flavor traits within the Principal components.

Items	PC1	PC2	PC 3	PC 4	PC 5	PC 6	PC 7	PC 8
Sourness	0.38	-0.31	0.27	-0.15	0.28	0.00	0.51	0.57
Bitterness	0.25	0.54	-0.35	-0.18	-0.07	0.51	-0.19	0.44
Astringency	0.42	0.18	0.11	0.88	0.00	0.00	0.00	0.00
Aftertaste-A	0.36	0.36	-0.27	-0.21	0.05	-0.79	0.00	0.00
Aftertaste-B	0.40	-0.14	-0.41	-0.11	0.30	0.31	0.31	-0.60
Umami	-0.37	0.35	-0.17	0.13	-0.31	-0.01	0.78	0.00
Aftertaste-U	-0.36	0.37	0.11	0.09	0.84	0.00	0.00	0.00
Saltiness	0.24	0.43	0.72	-0.29	-0.14	0.12	0.07	-0.34

In the spatial distribution of the PCA graphic, three clusters existed on the PCA map showed an easily visible separation of the different breeds with different colors, which could help us make the rapid identification on different breeds of beef. Legin et al (2003) found that the electronic tongue distinguished all wine samples of the same denomination and vintage [6]. Beullens et al. (2008) demonstrated that electronic tongues were very well suited to classify tomato cultivars based on their taste profile [26]. In addition, Silva et al. (2012) also reported that the electronic tongue could distinguish five different soybean cultivars through flavor attributes [27].

## Conclusion

In the present study, different breeds of beef had different chemical composition and flavor. PCA analysis showed an easily visible separation of different breeds of beef and proved that electronic tongue system TS-5000Z could make the rapid identification of different breeds of beef according to the flavor values. In addition, electronic tongue system TS-5000Z could be also used to predict the chemical composition based on the regression formula between chemical composition and flavor. Therefore, TS-5000Z would be used as a rapid analytical tool to evaluate the beef quality both qualitatively and quantitatively, based on flavor assessment, recognition and chemical composition according to its correlation with flavor.

## Supporting Information

S1 File The flavor and chemical composition of each meat sample. (XLSX)
